# Falso negativo en serología de VHC debido a interferencia por crioglobulinas

**DOI:** 10.1515/almed-2021-0004

**Published:** 2021-02-10

**Authors:** Gemma Recio Comí, Carmen Molina Clavero, Sandra Calabuig Ballester, Clara Benavent Bofill, Ester Picó-Plana, Carla Martín Grau, Cristina Gutiérrez Fornés, Mᵃ Teresa Sans Mateu

**Affiliations:** Laboratorio de Análisis Clínicos, Instituto Catalán de Salud (ICS)- Camp de Tarragona-Terres de l’Ebre, Hospital Universitario Joan XXIII, Tarragona, España; Laboratorio de Análisis Clínicos, Instituto Catalán de Salud (ICS)- Camp de Tarragona-Terres de l’Ebre, Hospital Verge de la Cinta Hospital de Tortosa, Tarragona, España

**Keywords:** virus de la hepatitis C, crioglobulinemia, interferencia

## Abstract

**Objetivos:**

Las crioglobulinas (CG) son proteínas séricas que precipitan en frío *in vitro*. Se sabe que las CG causan interferencias analíticas en diversos tipos de análisis clínicos, lo que se traduce en resultados incorrectos. Presentamos el caso de una paciente diagnosticada erróneamente de crioglobulinemia esencial debido a la interferencia de las CG en la serología del virus de la hepatitis C (VHC).

**Presentación del caso:**

Es el caso de una paciente edad avanzada edad con fallo renal agudo que requirió hemodiálisis urgente. Ante la ausencia de infecciones u otras patologías asociadas a la producción de CG, se estableció el diagnóstico de fracaso renal agudo causado por una crioglobulinemia esencial. Sin embargo, se obtuvo un resultado positivo inesperado al analizar la carga viral del VHC. De este modo, se sospechó de la presencia de una infección por VHC, con resultado falso negativo en la serología, causado por la interferencia *in vitro* de las CG. Esto se confirmó al repetir la serología en suero calentado a 37 °C. Finalmente, se estableció el diagnóstico de crioglobulinemia secundaria a infección por VHC.

**Conclusiones:**

Este caso evidencia que la presencia de CG en sangre puede interferir en la correcta interpretación de los resultados de diversos tipos de análisis clínicos. La identificación de las CG y el tratamiento previo del suero son cruciales a la hora de evitar resultados incorrectos y establecer un diagnóstico certero.

## Introducción

Los análisis clínicos son fundamentales a la hora de establecer un diagnóstico. Sin embargo, en algunos contextos, se pueden obtener falsos resultados debido a artefactos *in vitro*. Las paraproteínas son proteínas plasmáticas generadas masivamente en presencia de algunas patologías, lo cual puede influir en algunos parámetros clínicos [1]. Son conocidos los efectos *in vitro* de las crioglobulinas (CG) en el recuento de glóbulos con algunos analizadores. Se han observado recuentos erróneos de glóbulos blancos (GB), plaquetas y velocidad de sedimentación globular [2].

Las CG son un grupo de proteínas séricas con la propiedad física de precipitar reversiblemente a temperaturas inferiores a 37 °C. Las CG son producidas por la expansión de células B maduras clonales en patologías como el mieloma múltiple, los trastornos linfoproliferativos, infecciones crónicas o enfermedades autoinmunes. También pueden generarse en ausencia de patologías relevantes (crioglobulinemia esencial). Brouet et al. clasifican la crioglobulinemia en tres subgrupos, según la composición de las CG: tipo I (inmunoglobulinas monoclonales), tipo II (inmunoglobulinas monoclonales y policlonales) y tipo III (inmunoglobulinas policlonales). Los tipos II y III reciben el nombre de crioglobulinemia mixta, asociada al virus “de la hepatitis C (VHC)” [3]. La patogénesis de la crioglobulinemia aún no se ha descrito por completo, aunque la respuesta del sistema inmunológico podría jugar un papel fundamental. Se postula que la presencia de CG se debe a la viremia crónica y a la generación de factor reumatoide, desencadenadas por la exposición constante de las células B a complejos de antígenos de inmunoglobulinas [4].

Las CG mixtas se detectan en el 40% de los pacientes con infección por el VHC, y están asociadas a una mayor mortalidad y morbilidad. Los pacientes con VHC y CG tienen mayor riesgo de desarrollar cirrosis y presentar mayor grado de fibrosis que los pacientes sin CG. Además, la acumulación de CG en las paredes arteriales podría causar manifestaciones extrahepáticas como vasculitis, glomerulonefritis, artritis, púrpura, neuropatías y cerebritis [5].

## Caso clínico

Presentamos el caso de una mujer de 84 años derivada al Servicio de Nefrología afectada por fracaso renal agudo y edema pulmonar que requirió hemodiálisis de urgencia. La paciente acusó mareo y desorientación, aunque la presencia de patologías agudas o infecciosas se descartó mediante un TAC y punción lumbar. La paciente presentaba la tríada de Meltzer: lesiones purpúricas, artralgias y debilidad. Los análisis iniciales confirmaron daño renal: creatinina 2,44 mg/dL (valor de referencia (VR): 0,5–1 mg/dL), urea 198 mg/dL (VR: 18–46 mg/dL), potasio 6,1 mmol/L (VR: 3,5–5,1 mmol/L) y sodio 128 mmol/L (VR: 132–146 mmol/L). Se visualizó hematuria con eritrocitos dismórficos en el sedimento urinario, con presencia de proteinuria (+2 en la tira reactiva = 100 mg/dL). Los análisis también revelaron anemia normocrómica y normocítica, y un recuento elevado de leucocitos a expensas de neutrofília. La coagulación era normal. Los cultivos de sangre y orina fueron negativos. La radiografía torácica y el TAC abdominal fueron normales. La ecografía renal no mostró alteraciones morfológicas. No se pudo realizar una biopsia renal, a causa del mal estado general de la paciente. El estudio inmunológico mostró niveles bajos de C3 y C4 y valores elevados de factor reumatoide. No se detectaron anticuerpos antinucleares ni anticuerpos anticitoplasma de neutrófilos. La relación kappa/lambda libre y la cuantificación de IgM eran elevadas. En la electroforesis e inmunofijación de proteínas séricas se detectó una discreta banda en la fracción gammaglobulina. La presencia de una neoplasia hematológica se descartó mediante el análisis de aspirado de médula ósea. Se realizaron serologías virales, que fueron negativas, incluyendo la del VHC (ADVIA Centaur^®^ EIA HCV Assay, Siemens Healthineers). Otros resultados analíticos revelaron una crioglobulinemia de tipo II compuesta por IgG monoclonales e IgM monoclonales de tipo Kappa. La biopsia cutánea de las petequias mostró una vasculitis de vasos medianos. En ausencia de infecciones u otras patologías asociadas a la producción de CG, se estableció el diagnóstico de fracaso renal agudo causado por una crioglobulinemia esencial.

Siguiendo el protocolo de diálisis, se analizó la carga viral de VHC (COBAS 4800 HCV 120t, ROCHE Diagnostics^®^), con un resultado inesperado de 3.210.000 UI/mL. La paciente no presentaba síntomas, y los biomarcadores de función hepática no sugerían hepatitis. Se descartó la contaminación cruzada de muestras y el período ventana. En este punto, se sospechó de un falso resultado negativo en la serología de VHC, causado por el efecto de las CG *in vitro*.

Se repitió la serología del VHC en suero calentado a 37 °C durante 60 minutos para permitir la redisolución del posible crioprecipitado. Los anticuerpos del VHC resultaron ser reactivos, con un valor índice de 2,68 (reactivos si ≥1). Finalmente, se estableció un diagnóstico correcto de fracaso renal agudo causado por crioglobulinemia secundaria a infección por VHC. Se volvieron a analizar los niveles de inmunoglobulina, C3 y C4, factor reumatoide, electroforesis e inmunofijación de proteínas séricas con suero calentado a 37 °C, para evitar obtener resultados falseados por la interferencia de las CG.

El objetivo principal del tratamiento para la infección crónica de VHC es la erradicación del virus con terapia antiviral [6]. Por desgracia, no se pudo tratar a la paciente debido su incapacidad para tragar. El segundo objetivo es el tratamiento de los factores autoinmunes con corticoesteroides, Rituximab y/o plasmaféresis. La paciente se encontraba clínicamente inestable y no se le podía administrar ningún tratamiento agresivo. Ante la gravedad de la enfermedad y la avanzada edad de la paciente, se decidió no tratarla ni con Rituximab ni con plasmaféresis. En su lugar, se le administró terapia con corticoides para tratar las manifestaciones extrahepáticas, además de la hemodiálisis y la furosemida para la insuficiencia renal. El estado de la paciente empeoró, con hemorragia rectal severa (debido a las múltiples lesiones vasculares) y episodios de apnea. Se aplicaron medidas paliativas y, finalmente, la paciente falleció.

Hay que señalar que la ausencia de tratamiento para la infección por VHC no fue la causa del fallecimiento. La paciente no tenía síntomas ni alteraciones en los parámetros de función hepática (coagulación normal, transaminasas normales, hígado de tamaño y morfología normales, homogéneo, sin lesiones focales en el TAC). La paciente falleció debido a una vasculitis multisistémica secundaria al VHC, que no se pudo tratar con una terapia más agresiva.

## Discusión

La crioglobulinemia provoca falsos negativos en la PCR para la detección de ARN del VHC. En esta situación, la serología puede ser positiva, pero las partículas virales pueden quedar enmascaradas en el crioprecipitado [7]. Nuestro caso es justo el opuesto. Tini et al. describieron un caso similar en 2007, aunque solo pudieron detectar ARN del virus en el crioprecipitado [8].

Aquí presentamos un falso negativo en la serología de VHC causado por la interferencia de CG. Este caso evidencia que la presencia de CG en sangre puede interferir en la interpretación de los resultados de algunos análisis clínicos. Antes del descubrimiento de la infección por VHC, la mayoría de casos de crioglobulinemia eran descritos como crioglobulinemia esencial. Desde el momento en que se describió la asociación entre el VHC y las CG, el diagnóstico de crioglobulinemia esencial se ha vuelto muy poco frecuente. En este caso, la presencia de CG en sangre derivó en un diagnóstico erróneo de crioglobulinemia idiopática, en lugar de crioglobulinemia secundaria a VHC. Esto se podría explicar por la precipitación de las CG, que atrapan los anticuerpos del VHC del suero, llevando a un falso resultado negativo. Al precalentar el suero a 37 °C, se solubiliza el crioprecipitado, liberando los anticuerpos y permitiendo su detección que, en este caso, reveló una infección de VHC que no había sido diagnosticada hasta el momento.

Por lo general, los falsos negativos suelen ser más preocupantes que los falsos positivos. Dado el gran impacto clínico de la infección por VHC, un falso negativo en la serología supone un riesgo para el paciente y para la propia comunidad. Por un lado, un paciente sin diagnosticar no recibirá tratamiento antiviral. Por el otro, procedimientos médicos y analíticos como la hemodiálisis se podrían administrar al paciente sin tomar medidas preventivas, suponiendo un riesgo de transmisión nosocomial de la infección.

La realización de un análisis de alta sensibilidad es crucial a la hora de evitar falsos negativos. La sensibilidad de la técnica ADVIA Centaur^®^ EIA HCV descrita por Siemens es del 100% (95% CI). Se realizaron múltiples estudios para compararla con otras técnicas alternativas con marca CE como Architect^®^ Anti-HCV o Elecsys^®^ Anti-HCV. La concordancia general entre Advia Centaur^®^ Anti-HCV y los otros analizadores fue satisfactoria. La sensibilidad del análisis de Siemens es equivalente a las de las otras marcas y es adecuada para el análisis rutinario del VHC [9], [10].

Existen casos de falsas serologías negativas para otros virus por la interferencia de CG. También se han descrito casos de falsos positivos (por ejemplo, en infecciones por Chikunguña o sífilis) [11], [12]. Algunos casos de falsos negativos en serología de VHC pueden ser debidos al periodo ventana [13]. Los inmunoensayos enzimáticos pueden ser negativos en pacientes inmunodeprimidos y en pacientes que se someten a hemodiálisis [14]. En este caso, el periodo ventana se descartó y la paciente no estaba recibiendo tratamiento inmunosupresor cuando se realizaron las serologías.

## Conclusiones

Algunas paraproteínas séricas causan numerosas interferencias analíticas. Este caso demuestra la importancia de investigar las discrepancias en los resultados analíticos para establecer un diagnóstico certero. Dado el impacto clínico de la infección de VHC, un falso negativo en la serología supone un riesgo importante. Ante un resultado positivo de CG pero negativo en las serologías virales, proponemos la aplicación de un algoritmo que incluya la repetición de las serologías con suero precalentado a 37 °C durante 60 minutos para reducir la interferencia de las CG. La detección de ARN del VHC en combinación con la serología se postula como la manera más segura de evitar que una infección activa pase desapercibida en los análisis.
